# What do You Need to Get Male Partners of Pregnant Women Tested for HIV in Resource Limited Settings? The Baby Shower Cluster Randomized Trial

**DOI:** 10.1007/s10461-016-1626-0

**Published:** 2016-12-08

**Authors:** Echezona E. Ezeanolue, Michael C. Obiefune, Wei Yang, Chinenye O. Ezeanolue, Jennifer Pharr, Alice Osuji, Amaka G. Ogidi, Aaron T. Hunt, Dina Patel, Gbenga Ogedegbe, John E. Ehiri

**Affiliations:** 1 Global Health and Implementation Research Initiatives, School of Community Health Sciences, University of Nevada, Las Vegas, 4505 S. Maryland Parkway, Las Vegas, NV USA; 2 HealthySunrise Foundation, 8752 Castle Ridge Avenue, Las Vegas, NV 89129 USA; 3 Prevention, Education, Treatment, Training and Research-Global Solutions-PeTR-GS, Plot 25 Liberty Estate, Independence Layout Enugu, Enugu, 400001 Enugu State Nigeria; 4 School of Community Health Sciences, Lombardi Recreation Center, University of Nevada, Reno, MS-274, RM212, Reno, NV 89557 USA; 5 Center for Healthful Behavior Change, New York University Langone Medical Center, 550 1st Ave, New York, NY USA; 6 Department of Health Promotion Sciences, Mel and Enid Zuckerman College of Public Health, University of Arizona, 1295 N. Martin Ave., Tucson, AZ 85724 USA; 7 School of Community Health Sciences, University of Nevada, Las Vegas, 4505 S. Maryland Parkway, Box 454009, Las Vegas, NV 89154-4009 USA

**Keywords:** Male involvement, HIV testing, Prevention of mother-to-child transmission of HIV, Nigeria, Community-based research

## Abstract

Male partner involvement has the potential to increase uptake of interventions to prevent mother-to-child transmission of HIV (PMTCT). Finding cultural appropriate strategies to promote male partner involvement in PMTCT programs remains an abiding public health challenge. We assessed whether a congregation-based intervention, the *Healthy Beginning Initiative* (HBI), would lead to increased uptake of HIV testing among male partners of pregnant women during pregnancy. A cluster-randomized controlled trial of forty churches in Southeastern Nigeria randomly assigned to either the HBI (intervention group; IG) or standard of care referral to a health facility (control group; CG) was conducted. Participants in the IG received education and were offered onsite HIV testing. Overall, 2498 male partners enrolled and participated, a participation rate of 88.9%. Results showed that male partners in the IG were 12 times more likely to have had an HIV test compared to male partners of pregnant women in the CG (CG = 37.71% vs. IG = 84.00%; adjusted odds ratio = 11.9; p < .01). Culturally appropriate and community-based interventions can be effective in increasing HIV testing and counseling among male partners of pregnant women.

## Introduction

Research studies have shown that male involvement has a tremendous impact on women’s sexual and reproductive health. Male partner involvement has been shown to increase uptake of interventions designed for prevention of mother-to-child HIV transmission (PMTCT) and is associated with decreased infant HIV infection and increased HIV free survival among infants [1–4]. Male partner involvement is necessary to successfully implement the four-pronged comprehensive approach to HIV prevention advocated by the United Nations, which includes primary HIV prevention, supportive counseling for planned pregnancy for women living with HIV, access to antiretroviral therapy for the purpose of preventing mother-to-child transmission (MTCT) of HIV, and ongoing HIV care and support for HIV infected mothers and their infants, partners and families [5]. Couple HIV testing and counseling (HTC) remains an important entry point for male partner engagement in most forms of HIV prevention and care. Despite the promising findings of reduced MTCT with male partner involvement in antenatal care and the desire by women for their male partners’ participation, men’s involvement in antenatal care and uptake of HTC remains low in sub-Saharan Africa (SSA) [4, 6–9].

Rates of male partners attending at least one antenatal care visit have ranged from 1.8 to 32% in SSA and barriers to male partner participation in antenatal care and HIV testing have been identified at the individual, community, and health system levels [1, 4, 6, 7, 10–12]. Lack of awareness about HIV, a perception of low personal risk, mistrust between married couples, and fear of knowing one’s status are all identified barriers to men’s uptake of HIV testing and engagement in PMTCT [2, 6, 13–15]. Some of the community barriers in SSA are socio-cultural in nature and include gender norms that disapprove of male partners engaging in antenatal activities. This deep seated perception that antenatal activities are a woman’s responsibility has limited the role of male partners to only providing financial support during pregnancy [8, 12–14, 16–18]. Health system barriers include poor attitudes of service providers and the timing of antenatal services during work hours which makes it inconvenient for male partner participation [12, 13, 16, 17, 19–26].

Male partner approval is highly correlated with a woman’s use of HIV testing services [27–29]. Recent research in SSA has documented several effective interventions to promote male partner involvement, including such interventions as direct invitation to attend antenatal care-based HIV testing with their pregnant partners, offering HIV testing in bars, sending notification to partners of newly diagnosed HIV-positive women, mobile testing, and home-based testing [7, 9, 16, 20, 30–35]. The most successful interventions are those that use a combination of approaches that shift the burden of engaging the male partner from the woman to the community [36].

Nigeria has the highest burden of HIV infection in SSA. At the time of study implementation in 2013, Nigeria alone accounted for 17% of the estimated 1.3 million new HIV infections that occurred in SSA, including 26% of all new childhood infections that occurred among the 22 Global Plan priority countries [37]. Although 70% of men in Nigeria knew where to get an HIV test, only 10% had been tested for HIV and received the results in the past 12 months, and this rate was consistently lower in hard-to-reach rural communities compared to urban areas [38].

Nigeria has extensive networks of faith-based institutions, and faith plays a significant role in the social life of Nigerians [39]. In Southeastern Nigeria, the population is predominantly Christian (Catholic and Anglican) with church attendance nearing 90% [39]. Building on this foundation, we developed the Healthy Beginning Initiative (HBI), a culturally adapted, family-centered approach that relies on the widely distributed church-based networks to promote individual testing, tracking and linkage to care [40]. HBI was designed to remove many of the barriers to male partner testing and participation in antenatal care. The intervention provides male partners with (1) community encouragement to participate in antenatal care, (2) education about the antenatal risk of HIV, (3) integrated testing to reduce stigma, (4) testing at convenient locations and (5) free testing to reduce the economic burden of testing. HBI has been found to be effective for HIV testing and linkage to care for pregnant women and exposed infants [41, 42]. This paper reports the results of HBI on HIV testing among male partners of pregnant women during pregnancy, prior to delivery. We hypothesized that male partners of pregnant women randomized to the intervention group (IG) of HBI would have a higher rate of HIV testing compared to those randomized to the standard of care control group (CG).

## Methods

### Study Design, Church Recruitment and Randomization

A two-arm cluster randomized design was used to evaluate the effect of HBI on the rate of HIV testing among male partners of pregnant women. A detailed research protocol, church selection and sample size calculations for this study has been published elsewhere [40, 41]. In summary, a total of 40 churches in 40 communities in Enugu State, Southeast Nigeria, were randomly assigned 1:1 to either the IG (N = 20 churches) or CG (N = 20 churches). Self-identified pregnant women and their male partners 18 years and older who attended any of the study sites were eligible to participate. Participants in churches randomized to IG received health education and were offered an HIV test as part of an integrated, on-site laboratory test (hemoglobin, malaria, syphilis, HIV, sickle cell genotype, hepatitis B) during church organized baby showers prior to delivery. Participants in churches randomized to the CG were referred for HIV testing at their nearest healthcare facility, as is standard of care, during the church organized baby showers. The study was approved by the Institutional Review Board of the University of Nevada, Reno, and the Nigerian National Health Research Ethics Committee.

### Recruitment of Participants and Description of the Intervention

Recruitment began following randomization of the churches. Each Sunday, the priest (Catholic and Anglican) asked pregnant women and their male partners in the congregation to approach the altar for a prayer. He prayed for a healthy pregnancy, successful delivery, and encouraged pregnant women to seek care at a health facility during their pregnancy. He introduced HBI, the study team, and described the program’s objectives. The intervention included education about healthy pregnancy and integrated testing (hemoglobin, malaria, sickle cell genotype, HIV, hepatitis B, and syphilis), which reduced the concerns about stigma associated with HIV for participant recruitment. Pregnant women who consented could participate in the study even when the male partner was unavailable or chose not to participate. The baby showers and baby receptions were held at the churches and on Sunday to enhance participation, particularly of the male partners who worked during the week.

### Baby Shower (Before Infant Birth)

A baby shower was conducted one Sunday each month for pregnant women and their families and was similar for the CG and the IG with the exception of the intervention. Both groups received refreshments and participated in a gift exchange. During the baby shower, pregnant women and their male partners in the CG were referred to a local health facility for antenatal care and free HIV testing. The research team maintained direct contact with health facilities to confirm HIV testing and PMTCT completion.

The intervention received by the IG included an educational game show (The Game of Love) which provided information about healthy pregnancy habits in addition to HIV acquisition modes, and effective PMTCT interventions. Participants discussed healthy habits and important laboratory tests during pregnancy. Additionally, during The Game of Love, the male partner was asked to show a sign of love to his partner. The acts that the male partners chose to perform included feeding, carrying, kissing, and dancing. During the educational program, there was a connection made between performing an act of love and participating in integrated testing with their partner. Free integrated laboratory tests (hemoglobin, malaria, sickle cell genotype, HIV, hepatitis B, and syphilis) were offered to pregnant women and their male partners during the baby shower. During the development of the intervention, male partners were asked which tests should be integrated into the test offered along with HIV. Male partners wanted hemoglobin test included because they felt having a high hemoglobin level was a sign of strength. We included this test to increase motivation for male testing and participation. Additionally, the integrated testing was designed to reduce stigma associated with HIV-only testing. Women and their male partners identified as HIV-infected were linked to Prevention, Education, Treatment, Training and Research-Global Solutions (PeTR-GS), a President’s Emergency Plan for AIDS Relief (PEPFAR) supported, comprehensive program for HIV care. All counseling and follow-up HIV care took place at PeTR-GS facilities.

### Baby Reception (After Infant Birth)

One Sunday every two to three months, a baby reception was held for new births in the Church. The baby reception was attended by participants in both the CG and IG. Baby gifts and refreshments were provided, and participants completed a post-delivery questionnaire that offered an opportunity to ascertain and document HIV testing during pregnancy and pregnancy outcomes in addition to gather data used in the analysis. It also provided an opportunity for follow-up with women and their male partners who needed ongoing care post-delivery. Women and their male partners in the control group were offered free integrated HIV testing at the baby reception post-delivery.

### Assessment and Outcome Measures

The outcome measure was verified completed HIV testing for male partners of the pregnant women during pregnancy. Follow-up was conducted during the baby reception where post-delivery questionnaires were administered by the HT. The questionnaires were used to ascertain HIV testing during pregnancy in addition to gather data to identify predictors of HIV testing. Male-partners were asked to self-report HIV testing. Self-reported HIV tests were confirmed through the integrated laboratory at churches randomized to IG. Self-reported HIV tests in churches randomized to CG were confirmed at the health facility where the HIV tests reportedly took place. Only verified tests were used for analyses, and if we could not verify a test, it was considered as HIV-non-tested.

### Statistical Analysis

There were two important sample estimates for the study. First is N, the number of pregnant women and K, the number of churches, with the pregnant women nested within the K churches. A detailed sample size calculation and analysis plan has been described previously [40]. Our hypothesis test was for differences in two binomial proportions at follow-up. We applied the concept of Intention-to-Treat Analysis (ITT) for data analyses where we included all randomized subjects in the groups to which they were randomly assigned regardless of subsequent withdrawal from the program or deviation from the protocol. We included all subjects for part of the analysis and participants’ previous HIV test was treated as ITT stratagem of “last-observation-carried-forward” in order to reduce the effect of withdrawal. The χ^2^ statistic was used to assess differences in HIV-Test proportions. The Student’s *t* test was used to assess differences in continuous data. Multiple logistic regression was also used to analyze the binomial variable of HIV-tested and HIV-non-tested. Multiple logistic regression allowed for the incorporation of covariates and confounders at the individual level (age, education level, and previous HIV testing). Adjusted odds ratios (aORs) between HIV-tested and HIV-non-tested men were obtained by controlling the previously mentioned covariates and potential confounding factors. All above statistical significance tests were set as p-value <.05 and tests were 2-sided. Statistical Analysis Systems (SAS-9.4) was used for the analyses.

## Results

**Fig. 1:**
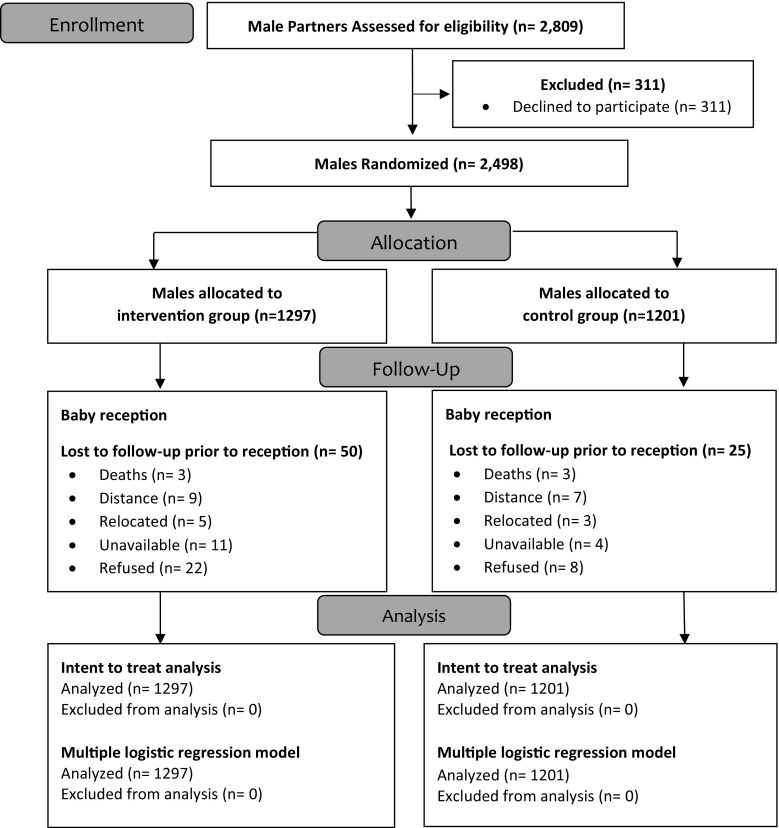
Healthy beginning initiative participant flow chart

Enrollment began in January, 2013 and was completed in September, 2013. Follow-up of enrolled participants ended in August, 2014. Of the 3047 women enrolled in HBI, 2809 were married or partnered from which 2498 males were enrolled and participated across 40 churches, a participation rate of 88.9%. Throughout the study a total of 75 participants were lost to follow-up (IG n = 50, CG n = 25) and did not complete the post-delivery follow-up questionnaire (See Fig. 1).

### Demographic Characteristics

Table 1 shows the demographic characteristics of the male partners who participated in the study. In general, participants in both control and intervention groups had similar demographic characteristics, including age, family size, and distance to the nearest health facility. However, some characteristics were significantly different. For example, participants in the control group were more likely to have tertiary level education (χ^2^ = 11.82, p = .003), more likely to have full time employment (χ^2^ = 6.5, p = .038), more likely to reside in urban areas (χ^2^ = 37.64, p < .001), and more likely to have previously tested for HIV (χ^2^ = 27.14, p < .001). We controlled for significant differences in subsequent analyses.

**Table 1:** Characteristics of participants

Characteristic		Total		Control group	%	Intervention group	%	χ^2^	p*
Total subjects			2498	1201		1297			
Age	Mean (STD)		38.32 (8.12)	38.48 (7.83)		38.17 (8.39)			.340
Age group									
16–24.9			64	30	2.51	34	2.64	2.49	.288
25–34.9			879	405	33.92	474	36.86		
35+			1537	759	63.57	778	60.5		
Education level									
None/primary			1044	490	40.90	554	43.18	11.82	.003*
Secondary			1102	517	43.16	585	45.60		
Tertiary			335	191	15.94	144	11.22		
Employment									
Full time			1503	759	63.51	744	58.49	6.53	.038*
Part time			611	276	23.1	335	26.34		
Unemployed			353	160	13.39	193	15.17		
Family size									
≤2			373	177	14.77	196	15.15	.01	.959
3–6			1785	859	71.7	926	71.56		
≥7			334	162	13.52	172	13.29		
Distance to health facility									
0–5 km			879	434	36.20	445	34.36	4.37	.225
5–10 km			929	458	38.2	471	36.37		
10–15 km			432	196	16.35	236	18.22		
15+ km			254	111	9.26	143	11.04		
Residency area									
Rural			1691	745	62.14	946	73.62	**37.64**	<.001*
Urban			793	454	37.86	339	26.38		
Self-reported previous HIV testing									
No			952	410	34.13	575	44.19	**27.14**	<.001*
Yes			1470	791	65.87	722	55.81		

* Results were considered statistically significant when p < .05

### HIV Testing Among Male Partners

Table 2 shows differences in rates of HIV testing and other related factors between control and intervention groups. Verified HIV testing rate among male partners were significantly higher in IG (84.00%) compared to CG (37.71%) (χ^2^ = 564.48, p < .001). Only self-reported previous HIV testing was significantly associated with higher HIV testing rates, indicating that men who previously had HIV testing were more likely to engage in HIV testing during this intervention (63.45 vs. 59.09%, p = .038).

**Table 2:** Confirmed HIV tests and predictors of HIV testing among males

		Total subjects (N)		Tested (N)	Rate (%)	χ^2^	p*
Confirmed HIV test	Control		1201	453	37.71	564.48	<.001*
	Intervention		1297	1089	84.00		
Age group	16–24.9		63	35	55.56	2.55	.279
	25–34.9		852	551	64.67		
	35+		1514	949	62.68		
Education level	None/Primary		1026	632	61.60	3.18	.204
	Secondary		1080	681	63.06		
	Tertiary		325	218	67.08		
Employment	Full time		1477	931	63.03	.34	.843
	Part time		593	376	63.41		
	Unemployed		346	213	61.56		
Family Size	≤2		366	238	65.03	.77	.679
	3–6		1746	1095	62.71		
	≥7		329	205	62.31		
Distance to Health Facility	0-5 km		862	523	60.67	5.07	.167
	5-10 km		908	572	63.00		
	10–15 km		425	278	65.41		
	15+ km		248	167	67.34		
Residency area	Rural		1652	1048	63.44	.58	.447
	Urban		781	483	61.84		
Ever tested for HIV (self-Reported)	No		985	582	59.09	4.31	.038*
	Yes		1513	960	63.45		

* Results were considered statistically significant when p < .05

### Adjusted Odds Ratio for Not Getting HIV Test Among Male Partners

Table 3 shows the adjusted odds ratios after controlling for demographic factors and other potential predictors for not having an HIV test among male partners of pregnant women. Male partners in intervention group were 12 times more likely to have a confirmed HIV test compared to male partners in control group after controlling for age, education, employment, area of residence, household size and a history of previous HIV testing (AOR: 11.67, 95% CI 9.4–14.4; p < .001). Having a tertiary school education and self-reported previous HIV testing were significantly associated with having an HIV test.

**Table 3:** Multiple logistic regression and adjusted odds ratios for determinants of HIV test among male partners

Variable	AOR	95% CI	p value
Confirmed HIV test			
Intervention group	11.96	9.63–14.79	<.001*
Age group			
35+ compared to 16–24.9	1.21	.77–2.67	.642
35+ compared 25–34.9	.98	.80–1.23	.608
Education level			
Tertiary compared to none/primary	1.53	1.12–2.08	.046*
Tertiary compared to secondary	1.53	1.11–2.12	.068
Working			
Full-time compared to part-time	1.03	.81–1.31	.830
Full-time compared to unemployed	1.12	.84–1.50	.490
Distance to healthcare facility			
0–5KM compared to 5–10KM	1.01	.75–1.37	.846
0–5KM compared to 10–15KM	.98	.79–1.23	.504
0–5KM compared to 15+ KM	1.16	.81–1.65	.390
Household size			
≥7 compared to ≤2	1.16	.87–1.55	.515
≥7 compared to 3–6 versus	1.16	.80–1.70	.617
Living area			
Rural compared to urban	.90	.72–1.21	.356
Self-reported previous HIV testing			
Yes compared to no	1.61	1.30–2.00	<.001*

* Results were considered statistically significant when p < .05

## Discussion

Findings from this study demonstrated that the intervention (i.e., culturally appropriate, congregation-based provision of education and onsite HIV testing to male partners of pregnant women during church organized baby showers for pregnant women) was more effective than standard care (i.e., referral to health facility to obtain education and HIV test), even though the male partners in the control group were exposed to part of the intervention which included a baby shower and reception. Male partners of pregnant women enrolled in the intervention group were 12 times more likely to have tested for HIV compared to male partners of pregnant women in the control group.

Exclusion of male partners during PMTCT programs reinforces the cultural notion that antenatal care and HIV/AIDS prevention are primarily a problem for women, and that responsibility for preventing transmission of HIV to their unborn babies is women’s sole responsibility. Nigeria is one of the 22 Global Plan priority countries that account for 90% of pregnant women living with HIV [37, 38, 43]. Despite improved efforts dedicated to the PMTCT, less than 20% of pregnant women in Nigeria were tested for HIV in 2013, only 27% of HIV-infected pregnant women received antiretroviral (ARV) therapy; and only 12% of HIV exposed infants received ARV prophylaxis for PMTCT [43–45]. The low rates of testing and treatment in Nigeria contributed to an estimated 58,000 HIV-infected infants in 2014 [44]. Finding new approaches to promote male partner involvement are necessary to realize the WHO/PEPFAR goal of eliminating new pediatric HIV infections [5].

Findings from this study support the existing body of evidence which shows that community-based programs to increase uptake of HTC are effective [6, 7, 16, 35, 36, 46]. Our intervention saw a male participation rate (88.9%) which outpaced other interventions to increase male participation in antenatal care in SSA that range between 16 and 35% [7, 16, 34]. Additionally, the rate of HIV testing among males was higher in both our IG (86.4%) and CG (38.2%) compared to the overall rate of HIV testing among males in Nigeria (23%) [47]. We believe the effectiveness of this intervention resulted mostly from its ability to address several individual, community, and health systems barriers that limit the involvement of male partners in antenatal care [6, 12, 14, 17, 47]. In cultures where programs associated with pregnancy and childbirth are traditionally regarded as women-only programs, male partners are unlikely to attend. To overcome this barrier, we involved male partners during the development of the intervention. Their suggestions during program development were critical to the success of the intervention. In a marked departure from most current practices, we implemented an approach that: (a) integrated HTC into an existing community-based, culturally-adapted and socially accepted institution; (b) chose to implement the intervention close to where the male partners live and congregate; (c) chose an integrated approach to education and testing that reduced the stigma associated with HIV-only testing; and (d) involved the male partners in specific activities. By implementing the program close to where male partners live, we reduced the barrier for them of having to take additional time away from work to attend antenatal care with their partners. Additionally, we gave male partners the role of presenting the baby shower gift to their pregnant partners during the baby shower. This provided a mechanism for the male partners to become actively involved in antenatal care outside of the health facility. The cultural environment in Nigeria is similar to other priority sub-Saharan Africa (SSA) countries which considers pregnancy to be a “women’s affair” and discourages “encroachment” by men [12, 14]. However, studies have shown that the majority of women want their male partners to participate in their pregnancy [8].

While interpreting the results of this study and contemplating its adaptation in other settings, it is important to consider the contextual factors that contributed to its success. For example, this program was successful because of the active and enthusiastic support of religious leaders and because it was conducted within an environment where religious institutions and their leaders have a strong influence in the community. For adaptation in other settings, influential leaders need to be identified and their support needs to be enlisted. Additionally, HBI was successful because HIV prevention fell within the cultural and spiritual values of religious leaders in Southeastern Nigeria. For example, many churches in Nigeria provide HIV counseling as part of pre-marital counseling of couples; some require mandatory proof of HIV testing as a condition for marriage [48]. For adaption of HBI in other geographic locations (i.e. mosques in northern Nigeria or Hindu temples in India), it must first be established that HIV prevention fits within the cultural and spiritual values of the influential leaders [49]. In Southeastern Nigeria, we identified churches as a place where people ‘congregate’. The place of ‘congregation’ will vary in other settings and this intervention might be adaptable to these settings. Additionally, HBI may be scaled up to include additional health screenings for other diseases such as diabetes and hypertension or mental health disorders, allowing HBI to serve as a valuable health resource in resource limited communities.

## Limitations

We conducted this study in Southeastern Nigeria where there is a high Christian church attendance rate. The generalizability of our findings to predominantly Muslim states in Nigeria, or other countries with lower church attendance rates may be limited. We only used verified HIV test. If test could not be verified (i.e. test was conducted at a health fair not associated with a health facility) it was counted as a non-test. This may have resulted in under counting of HIV tests. The questionnaire was written at a sixth grade reading level. We encountered a large number of participants who could not read English or the local language. We relied on the HT to administer the questionnaire because of the low rates of literacy. Other than verification of HIV testing, the rest of the data collected on the questionnaire were self-report. Face-to-face administration of the questionnaire and self-report may have introduced bias.

## Conclusion

This culturally adapted, family-centered, congregation-based intervention was effective in increasing HIV testing among male partners of pregnant women in Southeastern Nigeria. HBI or similar culturally appropriate platforms can be utilized to implement other health interventions designed for both communicable and non-communicable diseases in low-income countries with similar enabling contextual factors.
